# Retinal Glia Promote Dorsal Root Ganglion Axon Regeneration

**DOI:** 10.1371/journal.pone.0115996

**Published:** 2015-03-27

**Authors:** Barbara Lorber, Daniel J. Chew, Stefanie M. Hauck, Rachel S. Chong, James W. Fawcett, Keith R. Martin

**Affiliations:** 1 John van Geest Centre for Brain Repair, University of Cambridge, Cambridge, CB2 0PY, United Kingdom; 2 Research Unit Protein Science, Helmholtz Center Munich, German Research Center for Environmental Health, 85764 Neuherberg, Germany; 3 Cambridge NIHR Biomedical Research Centre, Cambridge, CB2 0PY, United Kingdom; 4 Wellcome Trust–Medical Research Council Cambridge Stem Cell Institute, Cambridge, United Kingdom; Hertie Institute for Clinical Brain Research, University of Tuebingen, GERMANY

## Abstract

Axon regeneration in the adult central nervous system (CNS) is limited by several factors including a lack of neurotrophic support. Recent studies have shown that glia from the adult rat CNS, specifically retinal astrocytes and Müller glia, can promote regeneration of retinal ganglion cell axons. In the present study we investigated whether retinal glia also exert a growth promoting effect outside the visual system. We found that retinal glial conditioned medium significantly enhanced neurite growth and branching of adult rat dorsal root ganglion neurons (DRG) in culture. Furthermore, transplantation of retinal glia significantly enhanced regeneration of DRG axons past the dorsal root entry zone after root crush in adult rats. To identify the factors that mediate the growth promoting effects of retinal glia, mass spectrometric analysis of retinal glial conditioned medium was performed. Apolipoprotein E and secreted protein acidic and rich in cysteine (SPARC) were found to be present in high abundance, a finding further confirmed by western blotting. Inhibition of Apolipoprotein E and SPARC significantly reduced the neuritogenic effects of retinal glial conditioned medium on DRG in culture, suggesting that Apolipoprotein E and SPARC are the major mediators of this regenerative response.

## Introduction

Axon regeneration in the adult central nervous system (CNS) is limited by several factors including axon growth inhibitory molecules such as chondroitin sulphate proteoglycans and growth cone collapsing myelin products along the pathway of growing axons [1], intrinsic changes in the growth capacity of maturing neurons [2] and lack of trophic support [3].

Several glial cell types have previously been shown to provide neuronal trophic support. These include peripheral nervous system (PNS)-derived Schwann cells, CNS-derived oligodendrocyte precursor cells, and olfactory ensheathing cells, a distinct glial cell type found in the olfactory system but sharing a common developmental heritage with Schwann cells [4], [5].

Recent studies have found a potent growth-promoting role for retinal astrocytes and Müller glia isolated from the mature rat CNS. Retinal glia were found to stimulate retinal ganglion cell neurite growth *in vitro* and to be important mediators of the repair response occurring in optic nerve regeneration and glaucoma models *in vivo* [6–8].

In the present study we sought to investigate whether retinal glia also exert a growth promoting effect outside the visual system. Interestingly, we found that retinal glia not only significantly enhanced neurite growth and branching of adult rat dorsal root ganglion neurons (DRG) in culture, but that transplantation of retinal glia also promoted axon regeneration in the dorsal root crush model *in vivo*. Furthermore, we found that retinal glia secrete Apolipoprotein E (ApoE) and secreted protein acidic and rich in cysteine (SPARC, also known as Osteonectin), which mediate the growth promoting effects of retinal glial conditioned medium.

## Materials and Methods

### Ethics Statement

Rats had unrestricted access to food and water, and were maintained on a 12 h light/dark cycle. Animal work was conducted in accordance with the U.K. Home Office regulations for the care and use of laboratory animals, the U.K. Animals (Scientific Procedures) Act (1986) and the ARVO Statement for the Use of Animals in Ophthalmic and Vision Research. All methods were approved by the University of Cambridge Animal Ethics Committee (Project licence 80/2423).

### Retinal glial cultures

Retinal glial cultures from either adult male Sprague-Dawley (Charles River, Margate, UK) or green fluorescent protein (GFP) transgenic Sprague-Dawley rats [9] were prepared as previously described [10]. One day after plating, non-attached neuronal cells were removed by gentle agitation and change of medium (DMEM containing 10% fetal calf serum; Invitrogen, Paisley, UK). Medium was subsequently changed every 2–3 days, resulting in highly purified retinal glial cultures, containing astrocytes and Müller glia, as previously described [6], [10]. After two weeks of culture, by which time glial cells had reached confluence, retinal glial conditioned medium from Sprague-Dawley cultures was prepared for use in DRG culture experiments and Mass spectrometry/Western blotting analysis. For this, cells were washed twice and incubated for 3 hours in serum-free DMEM medium. The medium was replaced with DMEM/F-12 nutrient mix (Invitrogen), conditioned for 24 hours and filtered (<0.2 μm) to remove non-adherent cells and debris. To prepare cells for *in vivo* transplantation, retinal glial cultures from GFP rats were washed with calcium/magnesium-free phosphate buffered saline (PBS, Invitrogen), incubated for 5 minutes in a 1x Trypsin-EDTA solution (Invitrogen), shaken to detach the cells, and DMEM containing 10% fetal calf serum was added. Cells were centrifuged, the supernatant removed and cells were resuspended in calcium/magnesium free PBS. This was repeated twice, with a final retinal glial cell concentration of 2 x 10^7^ cells/ml. For injection of dead glial cells, the cell suspension was boiled for 30 minutes at 80°C before use.

### DRG cultures

DRG were dissected from adult male Sprague-Dawley rats and incubated in Neurobasal-A medium (Invitrogen) containing 0.1% collagenase (Sigma, Poole, UK) (2 hours, 37°C, 5% CO_2_) and triturated in 1 ml of Neurobasal-A supplemented with L-glutamine (Invitrogen), B27 (Invitrogen), and gentamicin (Sigma). Cells were centrifuged through a 15% BSA gradient (900 rpm, 10 minutes), pelleted, and resuspended in supplemented Neurobasal-A medium. 750 DRG/well were cultured in either unconditioned medium (DMEM/F12 nutrient mix; control), retinal glial conditioned medium as prepared above, or retinal glial conditioned medium that had been pre-incubated with 1:100 goat anti-SPARC and/or goat anti-ApoE antibody (Santa Cruz, Dallas, USA) for 1 hour at room temperature before use [11], [12]. Furthermore, DRG were cultured in conditioned medium that had been pre-incubated with control antibody (goat IgG, Santa Cruz). DRG were cultured for 48 hours on sterile coverslips pre-coated with 100 μg/ml poly-L-lysine (Sigma) at 37°C in a humidified atmosphere containing 5% CO_2_. Three wells were plated per experimental condition and each experiment was repeated 3–4 times.

### Immunocytochemistry and quantification of DRG cultures

DRG were fixed with 4% paraformaldehyde at room temperature for 10 minutes and stained for rabbit anti-ßIII tubulin (1:1000, Covance, Maidenhead, UK) with Alexa 555 goat anti-rabbit (1:1000, Invitrogen) used as a secondary antibody, as previously described [13].

Images of 30 randomly selected DRG per experimental condition were captured, using an epifluorescence microscope (Leica, Wetzlar, Germany), and the length of their longest neurite was measured using the Leica Application Suite (LAS AF.1.8.0) program. For 3 wells/experimental condition, ßIII tubulin+ DRG were counted in 9 same-sized areas/well, averaged and the total number of DRG/well estimated.

Analysis of DRG neurite branching complexity was performed using the Sholl analysis semi-automated plug-in on image processing software Fiji [14]. The number of branch intersections with increasing radial distance from the soma was quantified at 10 micron intervals, up to the maximal length seen in control conditions for each set of experiments. Area under the curve was used to compare arborisation between groups.

Results were averaged over 3–4 experiments and expressed as mean + standard error of the mean (SEM). An unpaired two-tailed *t*-test (95% confidence interval [CI]), assuming equal variances, was used when comparison between two groups was needed. One-way ANOVA (95% CI) with Tukey multiple comparisons post hoc test (computed only if overall *P* < 0.05) was used when comparison of three or more treatment groups was required (GraphPad Prism; Graph-Pad Software Inc., La Jolla, Ca).

### Retinal glial transplantation and dorsal root crush

Surgeries were conducted in accordance with the U.K. Home Office regulations for the care and use of laboratory animals, the U.K. Animals Scientific Procedures Act (1986). Adult Sprague Dawley rats were anaesthetised with isoflurane (2% in 2L/min O_2_). A hemilaminectomy of the T12-L2 vertebral processes, overlying the L5 dorsal root entry zone, was accompanied by a unilateral L5 dorsal root crush 3mm from the spinal cord with fine forceps (3x for 5 seconds). Complete crush of the dorsal root was established by the crush area appearing as a translucent band after the crush. The tissue overlying the spinal cord was then closed in layers. Subsequently, a hemilaminectomy of the L5 lateral vertebral process exposed the L5 spinal nerve root and DRG. The epineurium was incised and glia were injected under the epineurium 1–2 mm distally to the L5 DRG into the spinal nerve side. For this, immediately prior to injection, GFP+ retinal glia were mixed with 0.4% 555 conjugated cholera toxin B subunit (CTB; Invitrogen). Subsequently, 1 x 10^4^ GFP+ retinal glia were injected in 1 μl calcium/magnesium free PBS, containing 0.2% 555 conjugated CTB (n = 7 rats for transplantation of dead glia; n = 8 rats for transplantation of live glia). Injections were performed over 10 minutes with a 30 gauge Hamilton syringe attached to a micro syringe pump [SYS-MICRO4, WPI, UK], and a gelfoam was placed over the injection site.

### Tissue processing and quantification of CTB labelled axons

Animals were transcardially perfused 14 days post-injury under terminal anaesthesia with 4% paraformaldehyde. L5 DRG, the adjacent dorsal root and spinal cord were removed, post-fixed, cryoprotected and embedded in OCT (Thermo Fisher Scientific, Cramlington, UK). Serial sections, at 8μm for L5 DRG and 18μm for L5 spinal cord, were cut with a cryostat and thaw-mounted onto Superfrost plus glass slides (VWR, Lutterworth, UK). Images of the dorsal horn were taken with an epifluorescence microscope in 4 sections per animal, under high magnification, and pixel intensity of CTB labelled axons was analysed with Image J (NIH, USA). Results are expressed as percentage difference in intensity of CTB labelling *versus* the dead glial transplantation control (set as 100% baseline). Results are expressed as mean ± SEM of 7–8 rats per condition for each of the experimental groups. The significance of intergroup differences was evaluated by an unpaired two-tailed *t* test (95% CI), assuming equal variances.

### Mass spectrometric analysis of conditioned medium

Conditioned medium from retinal glial cells was concentrated 5x on filter devices (Vivaspin, 3kDa cutoff) and then processed by filter aided sample processing as described [15]. Eluted and acidified tryptic peptides were loaded onto a trap column and then directly via HPLC (UltiMate nano-LC-system, LC Packings, Bensheim, Germany) into the mass spectrometer (LTQ Orbitrap XL, Thermo Fisher Scientific). Peptides and proteins were identified with Mascot search engine (Matrix Science Inc., Boston, USA) in the Ensembl Rat database with 10 ppm mass tolerance for peptides and 0.6 Da mass tolerance for fragments ions, carbamidomethylation set as fixed modification and oxidation of methionines and deamidations of asparagines and glutamines allowed as variable modifications. A Mascot-integrated decoy database search calculated a false discovery rate of <1%, using an ion score cut-off of 30 and a significance threshold of p<0.01 for all searches. Quantification was performed based on peptide intensities from the MS scans with Progenesis software (Nonlinear Dynamics, Newcastle, UK), as described [16].

### Western blotting of conditioned medium

Retinal glial conditioned medium was concentrated 20x using Amicon Ultra Centrifugal Filters (3 kDa cutoff; Millipore, Watford, UK). 3.5 μg of conditioned medium was loaded onto 4–12% NuPAGE Bis-Tris gels (Invitrogen), followed by transfer to polyvinylidene difluoride membranes (Millipore). Western blots were repeated twice using different conditioned medium samples. Membranes were incubated in 5% non-fat dry milk in PBS, containing 0.2% Tween 20, for 1 hour at room temperature. Subsequently, membranes were incubated overnight at 4°C with primary antibodies: rabbit anti-ApoE, 1:1000 (Abcam, Cambridge, UK); mouse anti-SPARC, 1:1000 (Thermo Fisher Scientific). Secondary antibodies (peroxidase-conjugated anti-rabbit or anti-mouse IgG; 1/10,000; Vector Labs, Peterborough, UK) were applied for 1 hour at room temperature. Chemiluminescence was detected using the Amersham ECL kit (Amersham, Buckingham, UK) and the Alliance 4.7 Western blot imaging system (Uvitec, Cambridge, UK).

## Results

### Retinal glia promote neurite growth and branching of DRG *in vitro* and DRG axon regeneration past the dorsal root entry zone *in vivo*

**Fig 1:**
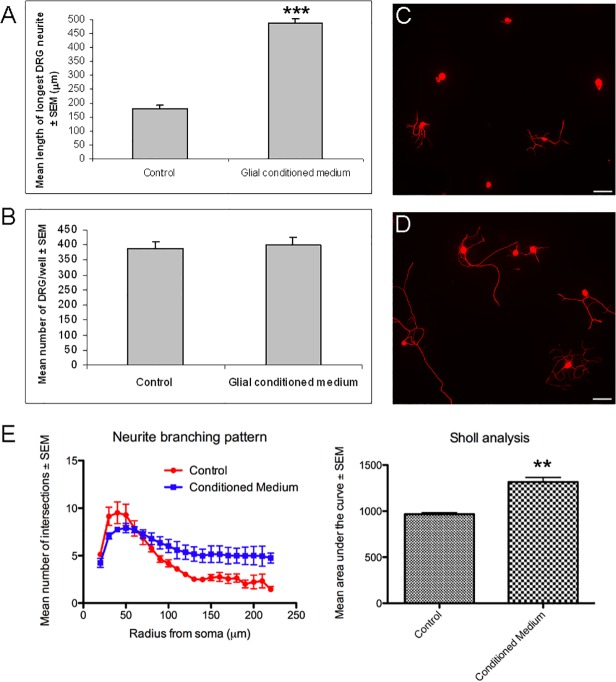
Retinal glial conditioned medium promotes neurite growth and branching of adult rat DRG in culture. Effects of retinal glial conditioned medium on (A) neurite growth and (B) survival of DRG in culture. Results consist of the means (± SEM) of 4 separate experiments. Photomicrographs of either (C) untreated DRG or (D) retinal glial conditioned medium treated DRG. (E) Neurite branching of adult rat DRG is significantly increased when cells are cultured in glial conditioned medium. Results consist of the means (± SEM) of 3 separate experiments. Significant differences are indicated by asterisk (P < 0.01 = **, P < 0.001 = ***). Adult rat DRG were cultured for 2 days. Scale bars: 100μm.

We initially tested whether conditioned medium derived from adult rat retinal glia, which contain astrocytes and Müller glia [6], [10], could enhance neurite outgrowth of adult rat DRG in culture. We found that retinal glial conditioned medium significantly enhanced neurite growth (P<0.001; Fig. 1A,C,D) of DRG after two days in culture compared to unconditioned medium, but did not affect the number of DRG present (Fig. 1B). Furthermore neurite branching was significantly enhanced in DRG cultures treated with retinal glial conditioned medium (P<0.01; Fig. 1E,C,D).

**Fig 2:**
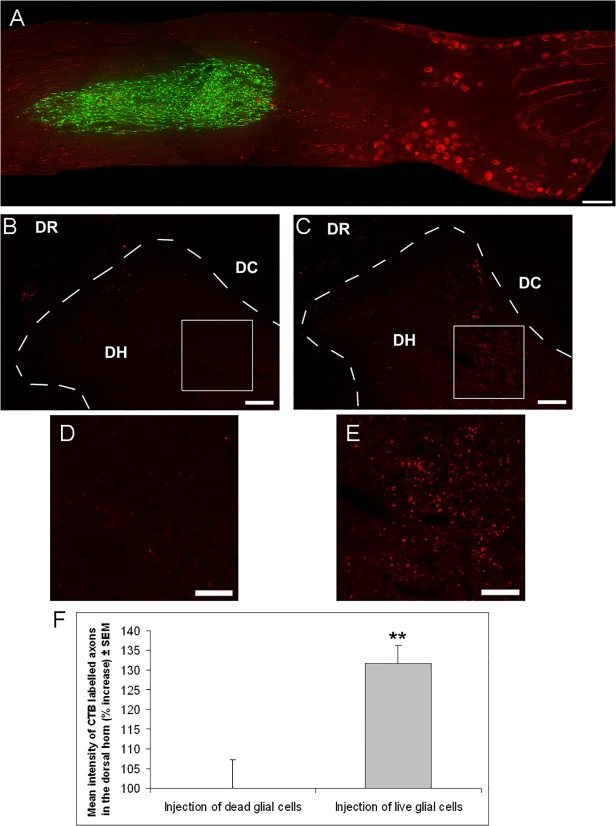
Transplantation of retinal glia significantly enhances DRG axon regeneration past the dorsal root entry zone. (A) Composite photomicrograph showing GFP+ retinal glia (green) that had been transplanted two weeks earlier 1–2 mm distally to CTB labelled L5 DRG neurons (red) into the spinal nerve side in an adult rat. (B,C) Low magnification images, of the dorsal root (DR), dorsal horn (DH) and dorsal column (DC) and (D,E) high magnification images of the dorsal horn, showing CTB labelled DRG axons, after transplantation of (B,D) dead retinal glia or (C,E) live retinal glia. (F) Quantification of the percentage increase in intensity of CTB labelled axons in the dorsal horn after transplantation of dead or live retinal glia in adult rats (n = 7 rats, dead glia; n = 8 rats, live glia). Animals in A-F were sacrificed two weeks after surgery. Significant differences are indicated by asterisk (P < 0.01 = **). Scale bars: (A) 200μm; (B,C) 100μm; (D,E) 50μm

We next tested if retinal glia promote DRG axon regeneration *in vivo*. For this, retinal glia derived from GFP transgenic rats were transplanted adjacent to L5 DRG neurons (Fig. 2A), as we wanted to ensure trophic support to the DRG without damaging them through the injection. DRG were CTB labelled (Fig. 2A) at the time of glial transplantation, and dorsal root crush was performed. After two weeks, axon regeneration past the dorsal root entry zone was assessed in the dorsal horn. As a control, dead retinal glial cells were transplanted.

Two weeks after dorsal root crush, only occasional CTB+ DRG axons had regenerated past the dorsal root entry zone in control (Fig. 2B,D), whilst transplantation of live retinal glia, resulted in significantly enhanced regeneration of CTB+ DRG axons into the dorsal horn (P<0.01; Fig. 2C,E,F).

### Retinal glia secrete ApoE and SPARC, which mediate the growth promoting effects of retinal glial conditioned medium

**Fig 3:**
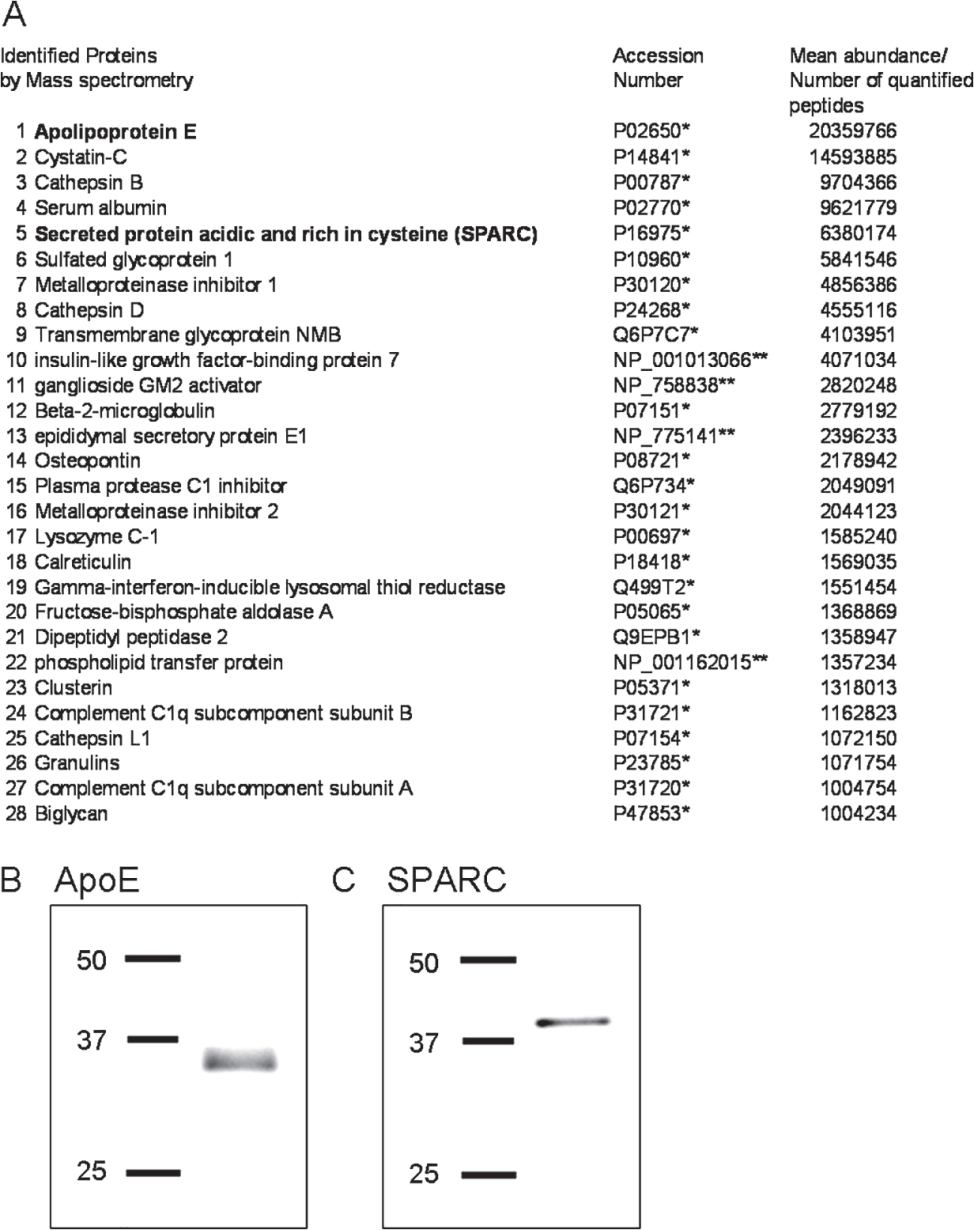
Mass spectrometric analysis of retinal glial conditioned medium shows that ApoE and SPARC are present in high abundance. (A) Mass spectrometric analysis of retina glial conditioned medium listing proteins (*Swiss/Prot Identifier, **RefSeq peptide Identifier) at mean abundance levels (n = 3 separate samples)/number of quantified peptides above 1000000. Western blots confirming the presence of (B) ApoE and (C) SPARC in retinal glial conditioned medium. Molecular weight markers in kDa.

To identify the factors that mediate the growth promoting effects of retinal glia, mass spectrometric analysis of retinal glial conditioned medium was performed. A total of 512 proteins were initially identified, of which 188 were found to contain a secretion signal. Of the secreted proteins, 28 were found to be present in high abundance (Fig. 3A; full list available on request). Among those 28 proteins, 3 had previously been implicated in modulating neurite outgrowth—ApoE, SPARC and Osteopontin [13], [17–20]. Osteopontin was shown to inhibit neurite outgrowth of DRG [20] and was therefore not considered further as a candidate mediating the neurite outgrowth promoting effects of retinal glial conditioned medium in the present study.

Both ApoE and SPARC were highly abundant in the retinal glial conditioned medium, with ApoE being the most abundant protein present (Fig. 3A). The presence of ApoE and SPARC in the retinal glial conditioned medium was further confirmed by western blotting (Fig. 3B: ApoE; Fig. 3C: SPARC).

**Fig 4:**
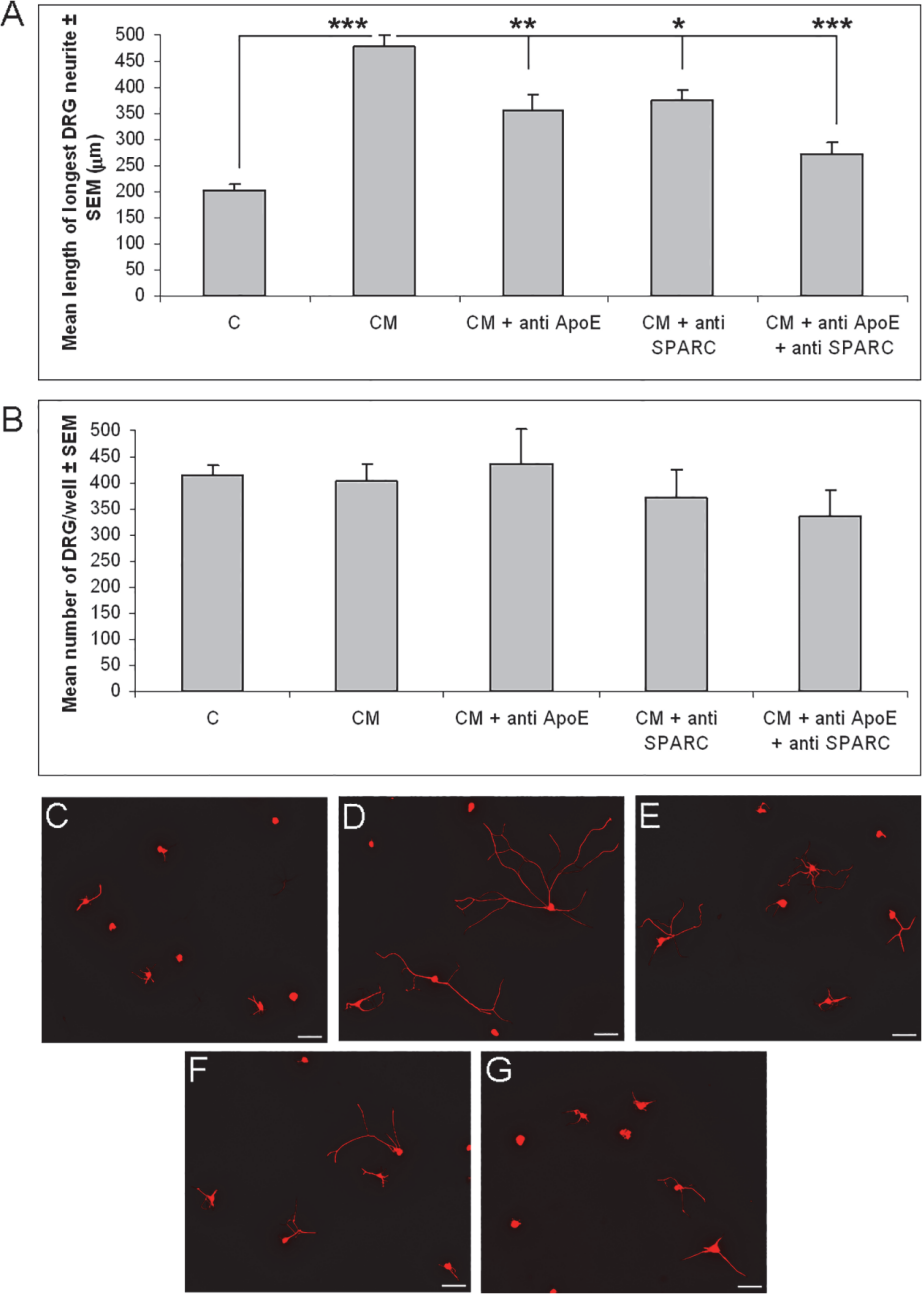
Inhibition of ApoE and SPARC significantly reduces the growth promoting effects of retinal glial conditioned medium. (A) Mean length of longest DRG neurite and (B) mean number of DRG after treatment with unconditioned medium, retinal glial conditioned medium or retinal glial conditioned medium which had been pre-incubated with anti-ApoE and/or anti-SPARC antibodies. Photomicrographs of DRG cultured in (C) unconditioned medium, (D) retinal glial conditioned medium, or conditioned medium that had been pre-incubated with (E) anti-ApoE, (F), anti-SPARC or (G) anti-ApoE and anti-SPARC antibodies. Significant differences are indicated by asterisk (P < 0.01 = **; P < 0.001 = ***). Results consist of the means (± SEM) of 3 separate experiments. Adult rat DRG were cultured for 2 days. CM = retinal glial conditioned medium; Scale bars: 100μm.

To test if ApoE and SPARC are indeed mediators of the growth promoting effects of retinal glial conditioned medium, we inhibited both factors, either singly or combined, with anti-ApoE and anti-SPARC antibodies [11], [12], and tested the conditioned medium on cultured DRG. Both anti-ApoE and anti-SPARC on their own significantly reduced the neurite growth promoting effects of retinal glial conditioned medium (anti-ApoE: P<0.01; anti-SPARC: P<0.05, Fig. 4A,C-F). Control IgG antibody had no significant effect on neurite growth (data not shown). Anti-ApoE and anti-SPARC combined reduced the growth promoting effects of retinal glial conditioned medium even further (P<0.001, Fig. 4A,C-G), though a small, albeit non-significant, growth effect remained. The number of DRG in culture was not significantly affected by any of the treatments (Fig. 4B).

## Discussion

We and others have previously uncovered a strong retinal ganglion cell axon growth promoting role for adult rat retinal glia [6–8]. In the present study we wanted to investigate whether this axon growth promoting ability of retinal glia could be translated to other parts of the nervous system.

We found that retinal glial conditioned medium strongly enhanced neurite growth and branching of adult DRG in culture, and chose the dorsal root crush model to investigate whether retinal glia also stimulate regeneration in the mature CNS outside the visual system. The dorsal root entry zone is a potent barrier to the regeneration of sensory DRG axons into the spinal cord after dorsal root crush [21]. We found that transplantation of live retinal glia resulted in a significantly enhanced number of CTB+ DRG axons regenerating into the dorsal horn compared to a dead glia transplantation control, showing that retinal glia indeed promote regeneration of sensory DRG axons into the spinal cord.

It has previously been shown that other glia may also promote neuronal axon growth, including PNS-derived Schwann cells, CNS-derived oligodendrocyte precursor cells and olfactory ensheathing cells, a distinct glial type that shares a common developmental heritage with Schwann cells [4], [5]. Our current findings that retinal glia promote DRG growth both *in vitro* and *in vivo* represent the first report to our knowledge of a strong growth promoting effect of glia derived from the mature CNS on DRG axon regeneration.

To identify the factors that mediate the growth promoting effects of retinal glia, mass spectrometric analysis of retinal glial conditioned medium was performed. Two factors found in high abundance, ApoE and SPARC, had previously been shown to modulate neurite outgrowth [13], [17–19] and were considered further as candidates mediating the growth promoting effects on DRG. Inhibition of ApoE and SPARC strongly reduced the growth promoting effects of retinal glial conditioned medium, though a small, albeit non-significant, growth effect remained. Since another 186 proteins, beside ApoE and SPARC, were identified in the conditioned medium, it is likely that some of these factors, either singly or in combination, contributed to the remaining growth effect. However, our results suggest that ApoE and SPARC, secreted by retinal glia, are the main mediators of the growth promoting effects. As Osteopontin, a DRG neurite growth inhibitory factor [20] was also found in high abundance in the conditioned medium it might be interesting in future studies to inhibit Osteopontin to see if the growth response facilitated by the glial conditioned medium can be further enhanced.

The present findings are in line with previous studies showing that retinal glia secrete ApoE [22], a lipoprotein that plays an important role in lipid metabolism in the nervous system [23], which has been found to promote growth of a host of neurons, including DRG [19], [24]. ApoE secreted by retinal glia is efficiently assembled into lipoprotein particles [22] and only in the lipoprotein form has it been shown to be neuroprotective both *in vitro* and *in vivo* [24], [25]. ApoE may mediate its growth promoting effect in the present study *via* receptors of the low density lipoprotein receptor family [26]. In agreement with this, other agonists of low density lipoprotein receptors (the Receptor Binding Domain of alpha-2-macroglobulin and the hemopexin domain of MMP-9) were shown to enhance DRG neurite growth *in vitro* and promote axonal regeneration after spinal cord injury *in vivo* [27].

Similarly, SPARC, a multifunctional extracellular matrix molecule, that was originally isolated from developing bone and referred to as osteonectin [28], has been previously shown to be expressed by retinal glia [29] and to be secreted by Schwann cells [18] and olfactory ensheathing cells [17]. SPARC was found to promote axon regeneration *in vitro* and *in vivo* either directly or indirectly through changing the activation state of Schwann cells *in vivo* to produce factors as laminin-1 and transforming growth factor β [17]. In line with this, SPARC was shown to act through receptors such as integrin β1 [30] and transforming growth factor β [31]. A direct receptor for SPARC has yet to be identified, though is likely to exist [18]. It is therefore possible that an indirect activation of Schwann cells by SPARC, and factors released by these cells, may also contribute in the present study to the observed regenerative effects. In addition, we found in our mass spectrometric analysis that retinal glia themselves secrete Laminin-1 and transforming growth factor β, albeit in very low abundance (data not shown).

In summary, this study is the first to show that glia from the adult CNS, retinal glia, can stimulate DRG neurite growth *in vitro* and regeneration of DRG axons past the dorsal root entry zone *in vivo*. Retinal glia were shown to secrete a host of factors, of which ApoE and SPARC appear to be major mediators of the retinal glial derived growth effects on DRG, though other factors, including factors derived from indirectly activated Schwann cells, may contribute to the observed growth effects. It will be interesting to test in future studies if SPARC and apolipoprotein E-containing lipoproteins, as ApoE secreted by retinal glia is assembled into lipoprotein particles [22], can mimic the growth promoting effects *in vitro* and *in vivo*.

In conclusion, this and previous studies in the visual system [6–8] show that retinal glia can have a potent growth promoting effect in different parts of the nervous system. It will be important in future studies to investigate their therapeutic potential for other CNS injury and disease models.
